# Mesenchymal stem cells release exosomes that transfer miRNAs to endothelial cells and promote angiogenesis

**DOI:** 10.18632/oncotarget.16778

**Published:** 2017-04-01

**Authors:** Min Gong, Bin Yu, Jingcai Wang, Yigang Wang, Min Liu, Christian Paul, Ronald W. Millard, De-Sheng Xiao, Muhammad Ashraf, Meifeng Xu

**Affiliations:** ^1^ Department of Pathology and Laboratory Medicine, University of Cincinnati Medical Center, Cincinnati, Ohio, USA; ^2^ Children's Nutrition Research Centre, Children's Hospital of Chongqing Medical University, Chongqing, China; ^3^ Department of Pharmacology and Cell Biophysics, University of Cincinnati Medical Center, Cincinnati, Ohio, USA; ^4^ Department of Preventive Medicine, School of Public Health, Guangzhou Medical University, Guangzhou, Guangdong Province, China; ^5^ Department of Pharmacology, University of Illinois at Chicago, Chicago, Illinois, USA

**Keywords:** exosomes, miRNA transfer, mesenchymal stem cells, angiogenesis, miR-30b

## Abstract

Mesenchymal stem cells (MSCs) have been found to benefit patients with a variety of ischemic diseases via promoting angiogenesis. It is also well established that exosomes secreted from MSCs deliver bioactive molecules, including microRNAs (miRs) to recipient cells. Therefore, we hypothesized that exosomes secreted from MSCs deliver miRs into endothelial cells and mediate angiogenesis. The pro-angiogenic stimulatory capacity of exosomes was investigated using tube-like structure formation and spheroid-based sprouting of human umbilical vein endothelial cells (HUVECs), and *in vivo* Matrigel plug assay. The secretion of pro-angiogenic miRs (pro-angiomiRs) from MSCs into culture medium and transfer of the miRs to HUVECs were confirmed using real-time quantitative PCR. Supplementation of the exosome secretion blocker GW4869 (10 μM) reduced the pro-angiomiRs in the MSC-derived conditioned medium (CdM^MSC^). Addition of exosomes isolated from CdM^MSC^ could directly 1) promote HUVEC tube-like structure formation *in vitro*; 2) mobilize endothelial cells into Matrigel plug subcutaneously transplanted into mice; and 3) increase blood flow inside Matrigel plug. Fluorescence tracking showed that the exosomes were internalized rapidly by HUVECs causing an upregulated expression of pro-angiomiRs in HUVECs. Loss-and-gain function of the pro-angiomiRs (e.g., miR-30b) in MSCs significantly altered the pro-angiogenic properties of these MSC-derived exosomes, which could be associated with the regulation of their targets in HUVECs. These results suggest that exosomal transfer of pro-angiogenic miRs plays an important role in MSC mediated angiogenesis and stem cell-to-endothelial cell communication.

## INTRODUCTION

It has been estimated that more than 500 million people will be benefited from various angiogenic therapies in the coming decades for treating ischemic diseases such as peripheral and coronary vascular disease [1], cerebral infarction [2], and critical limb ischemia [3]. Angiogenesis is a complex biological process involving interactions between vascular cells and the extracellular environment. Cell-based pro-angiogenic therapies have been increasingly utilized in the treatment of ischemic diseases [4, 5]. Consequently, stem cells have been extensively used to experimentally treat ischemic diseases including myocardial infarction [6, 7] and stroke [8, 9]. Mesenchymal stem cells (MSCs) have been recognized as a promising treatment option with the potential to generate a variety of useful cell-based interventions [10, 11] and pro-angiogenic therapies [12, 13]. Indeed, both *in vitro* and *in vivo* models have shown that MSCs can increase endothelial cell growth and enhance new blood vessel formation [14], as a result of paracrine effects that are considered as the predominant mechanism in addressing tissue damage [15]. We previously demonstrated that conditioned medium (CdM) of MSCs promoted post-infarction angiogenesis in ischemic myocardium and global heart function recovery [16]. However, the exact molecular mechanisms responsible for these beneficial paracrine effects of MSCs have not been identified.

Exosomes are cell-derived vesicles (diameter 30–100 nm) that exist in almost all biological fluids including blood, urine, saliva, cerebrospinal fluid, and cell preconditioned medium [17, 18]. They are initially formed by fusion of a multi-vesicular body with a plasma membrane, or released directly from the plasma membrane [17, 19]. Exosomes shuttle mRNAs, miRs, and other molecular constituents to achieve cell-to-cell communication, and modulate the function of recipient cells [20]. However, exosomes contents vary from different cell types, pathological conditions and by preconditioning or genetic manipulation of the parent MSCs [21, 22], which might cause completely inversed fate of target cells.

Most recently, the existence of miRs in exosomes has been reported [23–25], suggesting that exosomes may serve as a vehicle for miR transfer and mediate intercellular communication [26]. MiRs, a class of small non-coding RNAs (containing about 18–22 nucleotides), regulate gene expression on the posttranscriptional level by binding to specific mRNA and inducing their degradation and/or translational inhibition [27]. MiRs are recognized to participate in a wide range of biological and pathological processes including the cell cycle, hematopoiesis, neurogenesis, aging, cancer, and cardiovascular disease [28]. Evidence has suggested that miRs are key regulators of endothelial cell function and are especially important modulators of angiogenesis [29]. For instance, it has been reported that miR-424 promoted angiogenesis *in vitro* and in a mouse model by targeting cullin 2 [30]. miR-30 family targeted DLL4 in endothelial cells to promote angiogenesis [31]. The present study was designed to investigate whether MSC-derived exosomes shuttle various pro-angiogenic miRs and transfer these miRs to endothelial cells resulting in promoting angiogenesis.

## RESULTS

### Pro-angiogenic capacity of conditioned medium derived from MSCs

**Figure 1:**
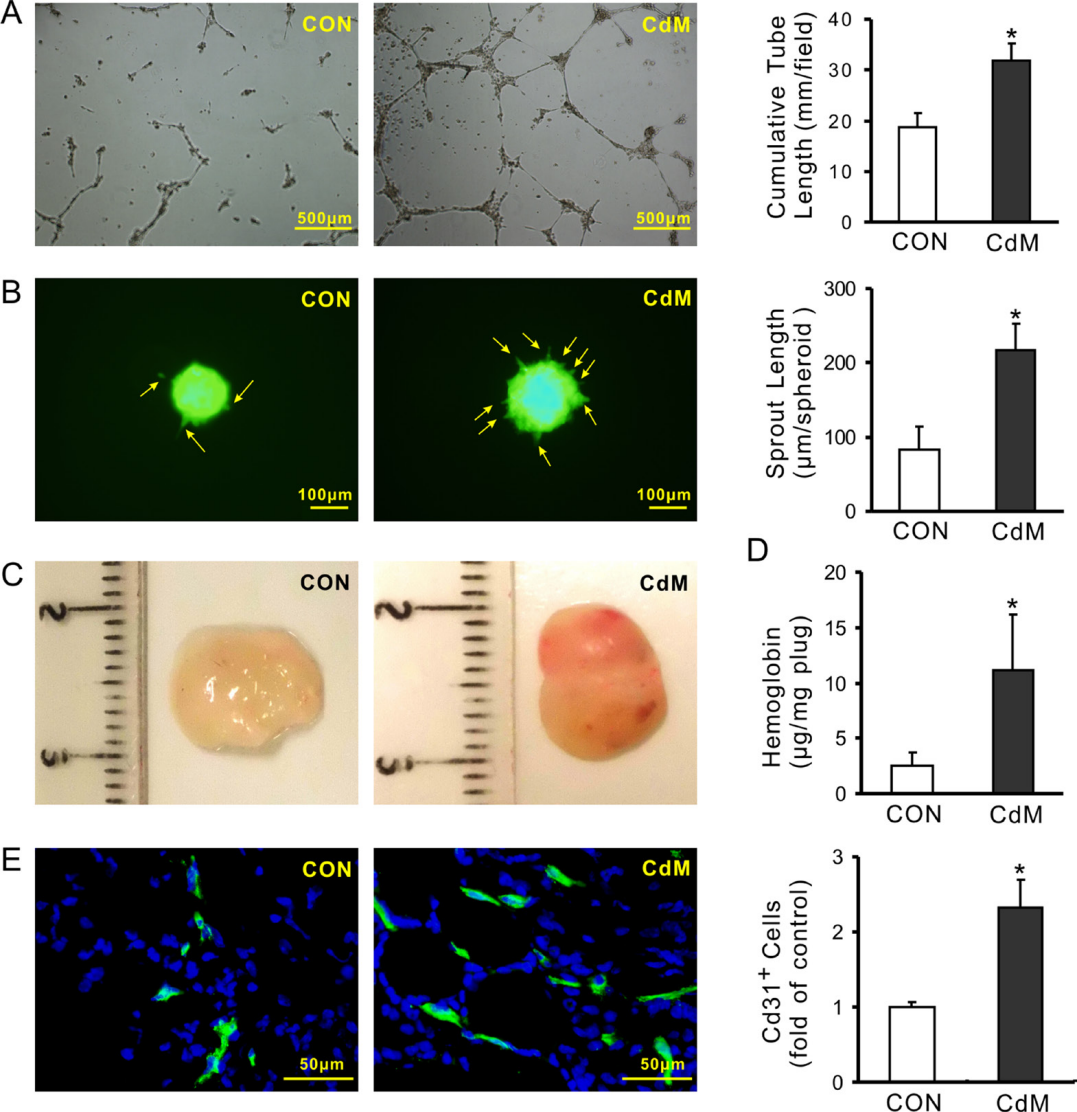
CdM derived from MSCs promotes angiogenesis (**A**) Representative images of capillary-like structures and quantitative analysis of the total tube length (4× magnification microscopic fields); (**B**) Representative images of HUVEC spheroids sprouting and quantitative analysis of the cumulative sprout length per spheroid; (**C**) Representative gross look of Matrigel plugs which were implanted subcutaneously in mice for 14 days; (**D**) Hemoglobin content in the Matrigel plugs; (**E**) The neovasculature in Matrigel was visualized by immunofluorescence staining of CD31. Quantification of the CD31-positive cells in Matrigel plugs were determined by pixel density (*P < 0.05 *vs* CON).

MSCs line C3H10T1/2 cells were purchased from ATCC (Manassas, VA, USA). MSCs adhered to the surface of plastic culture dishes and exhibited a spindle-shaped fibroblast-like morphology as shown in the Supplementary Figure 1. The pro-angiogenic capacity of CdM obtained from these cells (CdM^MSC^) was assessed using tube-like structure formation, spheroid-based sprouting of HUVECs and *in vivo* Matrigel plug assay. The cumulative tube length was significantly longer (31.80 ± 3.37 mm/field) in HUVECs treated with CdM^MSC^ compared to those treated with control medium (18.69 ± 2.83 mm/field) following culture for 16 h (Figure 1A). Sprout length per spheroid in HUVECs treated with CdM^MSC^ for 16 h was significantly longer (216.67 ± 36.29 μm/spheroid) than that treated with control medium (82.66 ± 32.23 μm/spheroid) (Figure 1B). The effect of CdM^MSC^ on endothelial cell invasion and hemoglobin concentration in Matrigel plug was investigated following subcutaneous injection of Matrigel into C57BL6 mice. The Matrigel plug contained CdM^MSC^ had a red gross appearance after transplanting for 14 days (Figure 1C). The hemoglobin content (a sign of increased new vessel formation) was significantly increased in the plugs containing CdM^MSC^ (11.14 ± 5.01 μg/mg plug) compared to the Matrigel plugs without CdM^MSC^ (2.48 ± 1.19 μg/mg plug) (Figure 1D). The neovasculature visualized by immunofluorescence staining of CD31 indicated that the number of CD31 positive cells in the plugs containing CdM^MSC^ was significantly higher than that without CdM^MSC^ (Figure 1E).

### miRs secreted from MSCs transfer to HUVECs

The expression of 26 most commonly acknowledged pro-angiogenic miRs (pro-angiomiRs) in CdM^MSC^ was quantified using real-time PCR before and after adding into HUVEC culture. The expression of miR-424, miR-30c, miR-30b, and let-7f in conditioned medium was significantly reduced after adding into HUVECs culture for 48 h, indicating that extracellular miRs, derived from MSCs, transferred into HUVECs. Meanwhile, the expression of miR-21, miR-10a, miR-126, miR-10b, miR-19a, miR-19b was significantly increased after adding into HUVECs culture, suggesting that HUVECs might release these miRs (Table 1). The expression of other miRs was either very low in CdM^MSC^ or the change was not significant (Supplementary Table 1).

**Table 1:** The expression of pro-angiogenic miRNAs in CdM^MSC^ after adding into HUVECs culture for 48 hours

Downregulated			Upregulated		
miRNA	CdM^MSC^ 2^(−ΔCt)^	CdM^MSC^ with HUVECs 2^(−ΔCt)^	miRNA	CdM^MSC^ 2^(−ΔCt)^	CdM^MSC^ with HUVECs 2^(?−ΔCt)^
miR-424**^#^**	44.965 ± 5.542	10.725 ± 1.795*	miR-21	89.021 ± 9.117	187.956 ± 27.620*
miR-30c	6.420 ± 0.623	0.572 ± 0.140*	miR-10a	0.435 ± 0.040	10.160 ± 0.985*
miR-30b	5.877 ± 0.692	0.133 ± 0.012*	miR-126	0.045 ± 0.014	6.988 ± 0.933*
let-7f	4.592 ± 0.245	0.153 ± 0.003*	miR-10b	0.008 ± 0.002	5.869 ± 0.442*
			miR-19a	1.623 ± 0.063	3.380 ± 0.316*
			miR-19b	1.540 ± 0.116	2.950 ± 0.225*

(*P < 0.05 *vs* CdM^MSC^).

^#^The mouse homologue of miR-424 sequence from human is miR-322-5p.

**Figure 2:**
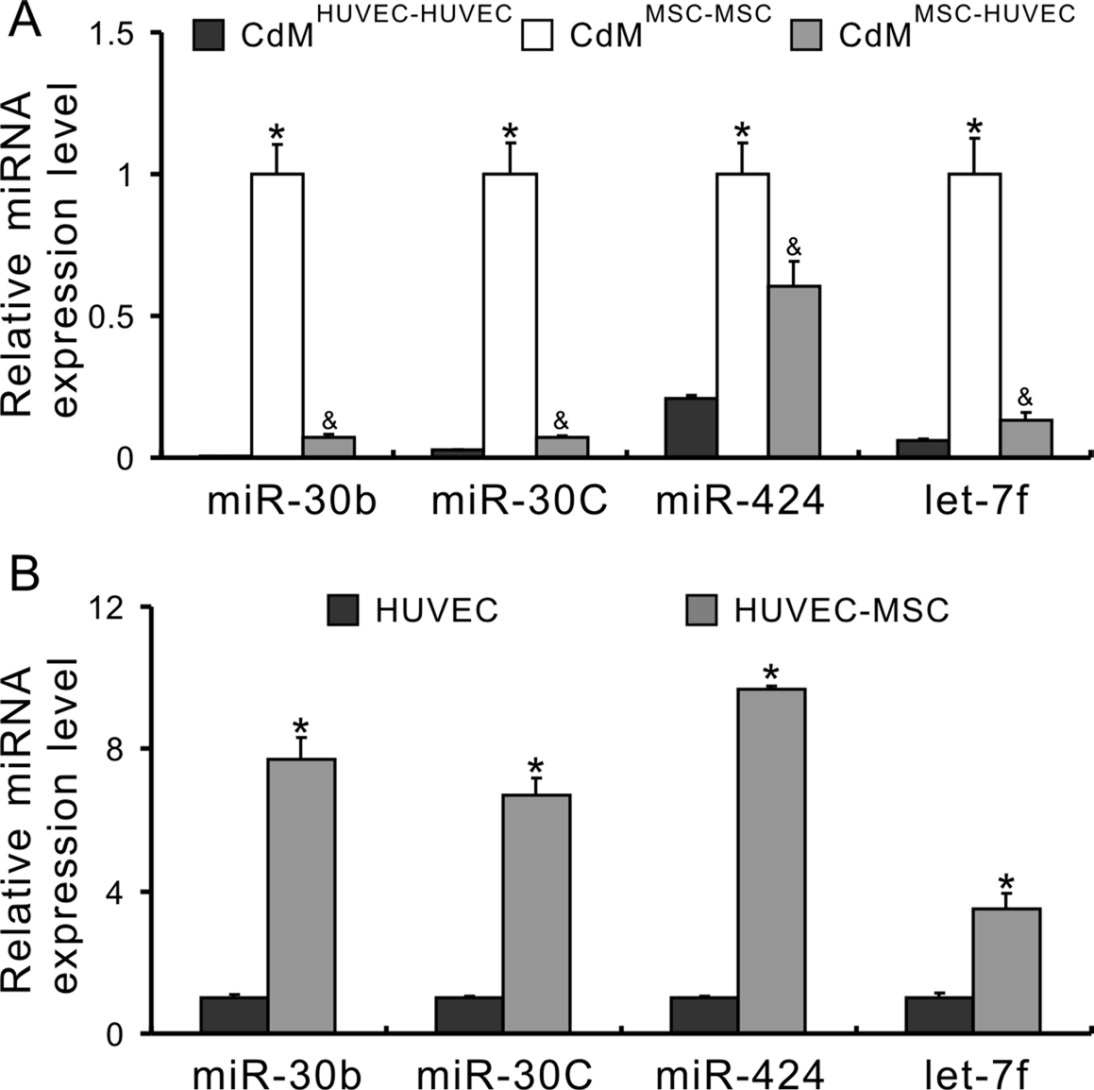
miRs secreted from MSCs transfer to HUVECs (**A**) miRNA expression in the CdM of non-contact cell co-culture system (*P < 0.05 *vs* CdM^HUVEC-HUVEC^; ^&^*P* < 0.05 *vs* CdM^MSC-MSC^, respectively); (**B**) The expression of miRs in HUVECs after co-culture with MSCs (*P < 0.05 *vs* HUVEC alone).

Next, the transfer of miRs between MSCs and HUVECs was determined using a non-contact co-culture system. The CdM was collected from MSC-HUVEC co-culture (CdM^MSC-HUVEC^) and its controls, MSC-MSC co-culture (CdM^MSC-MSC^) and HUVEC-HUVEC co-culture (CdM^HUVEC-HUVEC^), respectively. The expression of miR-30b, 30c, 424 and let-7f in CdM^HUVEC-HUVEC^ was very low and in CdM^MSC-MSC^ was very high. However, the expression of these miRs in CdM^MSC-HUVEC^ was significantly lower compared to those in CdM^MSC-MSC^, indicating that these miRs transferred into HUVECs (Figure 2A). Moreover, the expression of these miRs in HUVECs co-cultured with MSCs was significantly higher than in those without co-cultured with MSCs (Figure 2B), directly demonstrating a transfer of these miRs into HUVECs.

### Exosomes derived from MSCs deliver pro-angiomiRs and promote angiogenesis

**Figure 3:**
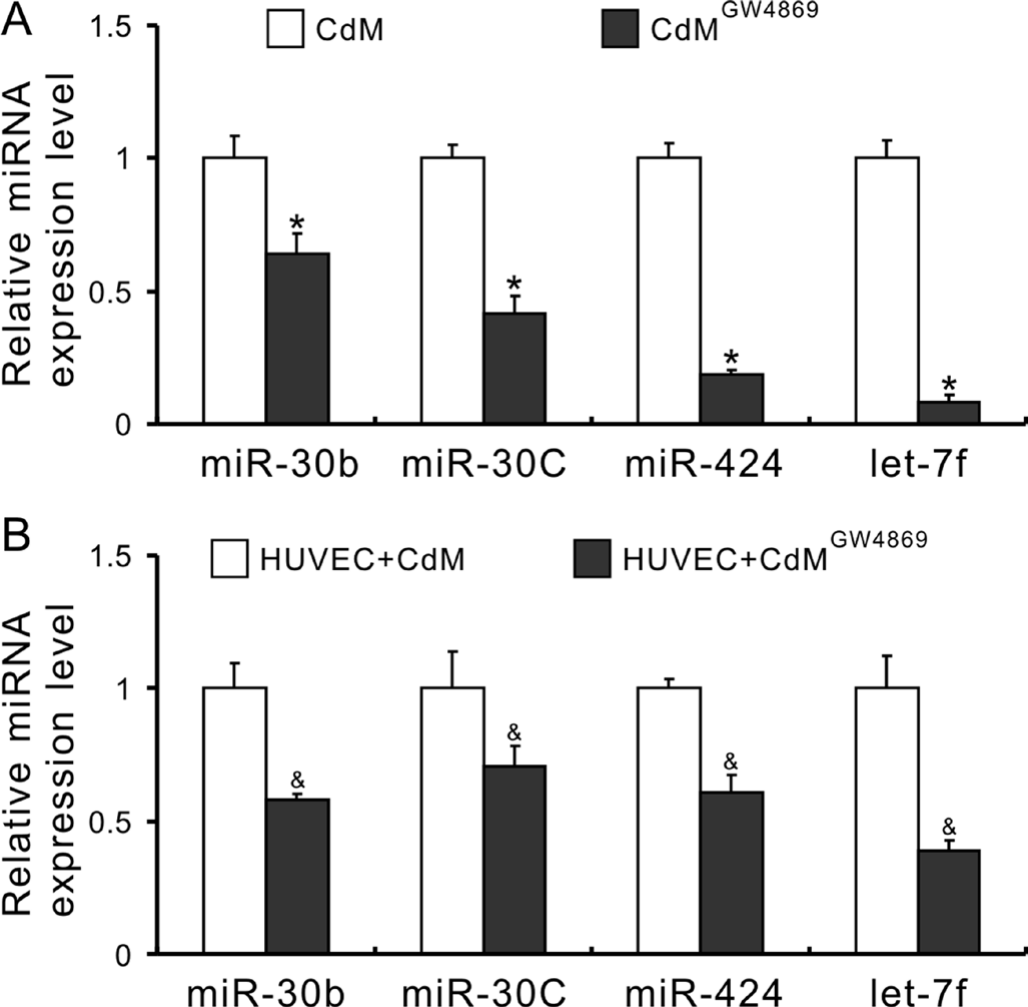
Exosomes mediate the transfer of miRs from MSCs to HUVECs (**A**) The expression of miRs in CdM (*P < 0.05 *vs* CdM^MSC^); (**B**) The expression of miRs in HUVECs treated with CdM (^&^*P* < 0.05 *vs* HUVEC + CdM^MSC^).

**Figure 4:**
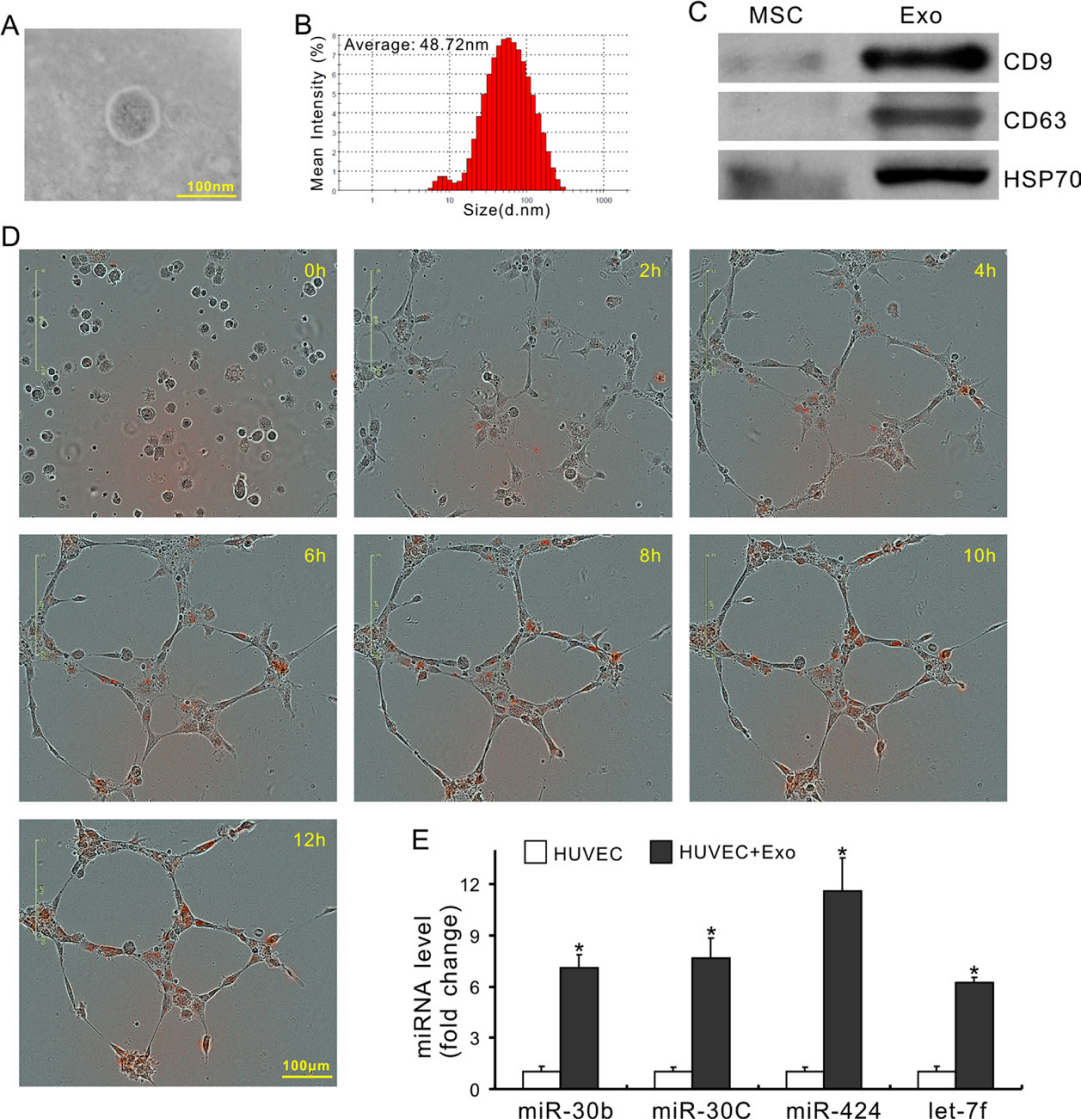
Characterization of exosomes and their involvement in miRs transfer (**A**) Morphology of exosomes under transmission electron microscopy; (**B**) The exosome size was measured using a Zetasizer Nano instrument. (**C**) The expression of CD9, CD63 and HSP70; (**D**) Representative images of time-lapse internalization of PKH26-labled exosomes (red) in HUVECs. (**E**) miR expression in HUVECs following exosome treatment (*P < 0.05 *vs* HUVEC+ BSA).

To investigate whether exosomes mediated miRs transfer, the expression of miRs in CdM was measured after MSCs were treated with 10 μM GW4869 (an exosome release inhibitor) for 48 h [32]. As shown in Figure 3A, the levels of miR-30b, -30c, -424, and let-7f in the CdM collected from MSCs treated with GW4869 (CdM^GW4869^) were significantly decreased compared with CdM obtained from control MSCs. In addition, the expression of these miRs in HUVECs treated with CdM^GW4869^ for 48h was also significantly decreased compared to those treated with CdM^MSC^ (Figure 3B), indicating that exosomes mediated miR transportation between MSCs and HUVECs. Exosomes, isolated from CdM^MSC^, exhibited the characteristic round morphology with heterogeneous size under transmission electron microscope (Figure 4A). The average size of exosomes was 48.72 ± 2.7nm according to the results of dynamic light scattering (Figure 4B). The expression of CD9, CD63, and HSP70 was significantly higher in exosomes compared to their parent MSCs (Figure 4C). The internalization of exosome pre-labeled with PKH26 by HUVECs was recorded using IncuCyte ZOOM Live Content Imaging System every 2 h for 12 h. The PKH26 red fluorescence intensity increased with the passage of time and achieved its maximum after exosomes were added into HUVECs culture for 10 h (Figure 4D). The expression of miR-30b, 30c, 424, and let-7f in HUVECs treated with exosomes for 24 h was significantly increased compared to those treated with BSA (Figure 4E).

**Figure 5:**
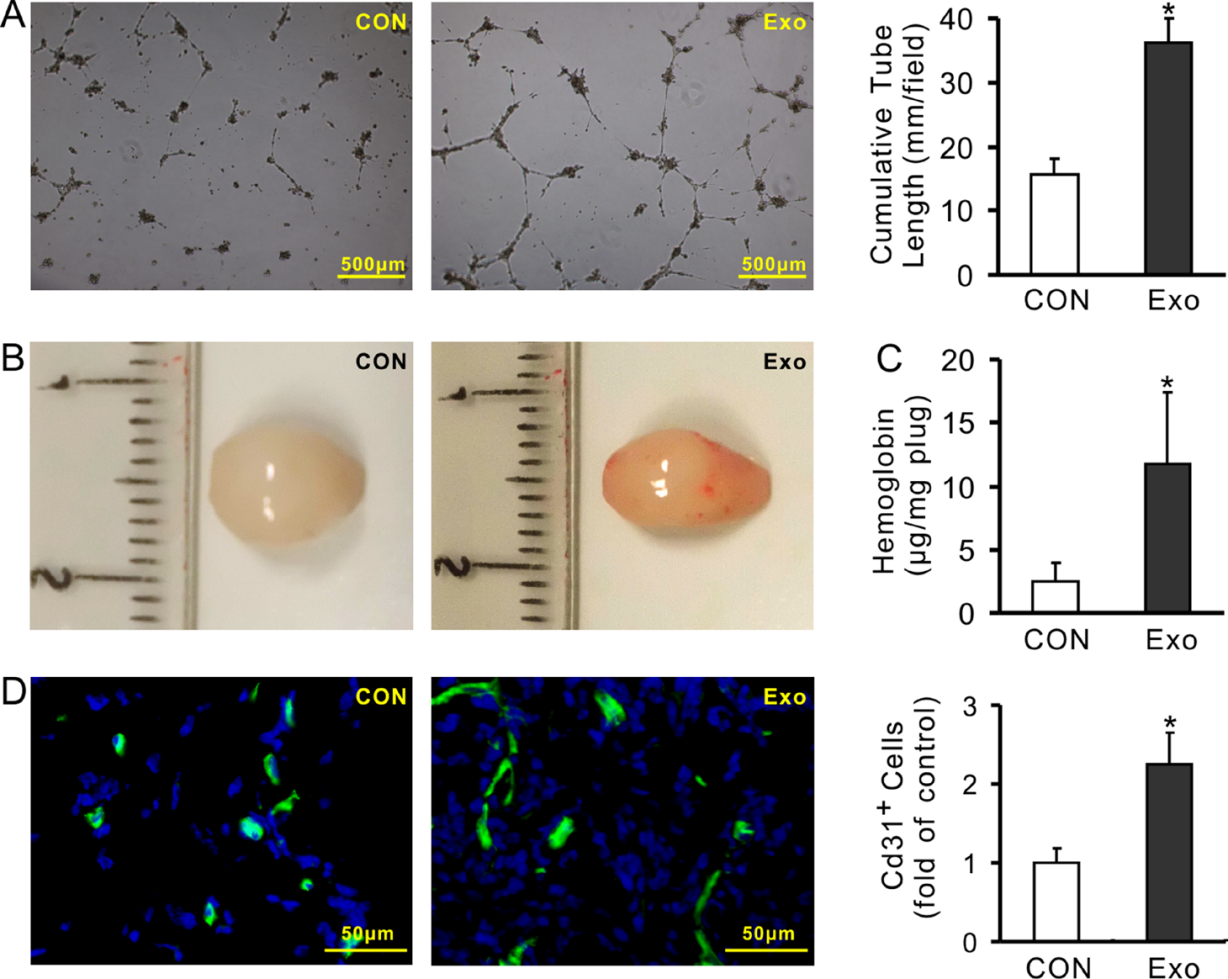
Exosomes derived from MSCs promote angiogenesis (**A**) Representative images of tube-like structures and quantitative analysis of the total tube length (4× magnification microscopic fields); (**B**) Representative gross look of Matrigel plugs; (**C**) Hemoglobin content in the Matrigel plugs; (**D**) Immunofluorescence staining of CD31 in the sections of Matrigel plugs and quantification of the CD31-positive cells (*P < 0.05 *vs* CON).

The pro-angiogenic capacity of exosomes was examined *in vitro* and *in vivo*. The cumulative tube length was significantly increased in HUVECs treated with exosomes (100μg/ml) for 16 h (36.24 ± 3.65 mm/field) compared to that treated with BSA in the same protein amount (15.73 ± 2.44 mm/field) (Figure 5A). The Matrigel plug test showed a more red appearance in those containing exosomes (100μg) after being transplanted for 14 days than those without exosomes (Figure 5B). The hemoglobin concentration in the plugs containing exosomes was significantly higher (11.76 ± 5.61 μg/mg plug) than those without exosomes (2.54 ± 1.45 μg/mg plug) (Figure 5C). The immunofluorescence staining showed that the number of CD31 positive cells in the plugs containing exosomes was also significantly higher than those without exosomes (Figure 5D).

**Figure 6:**
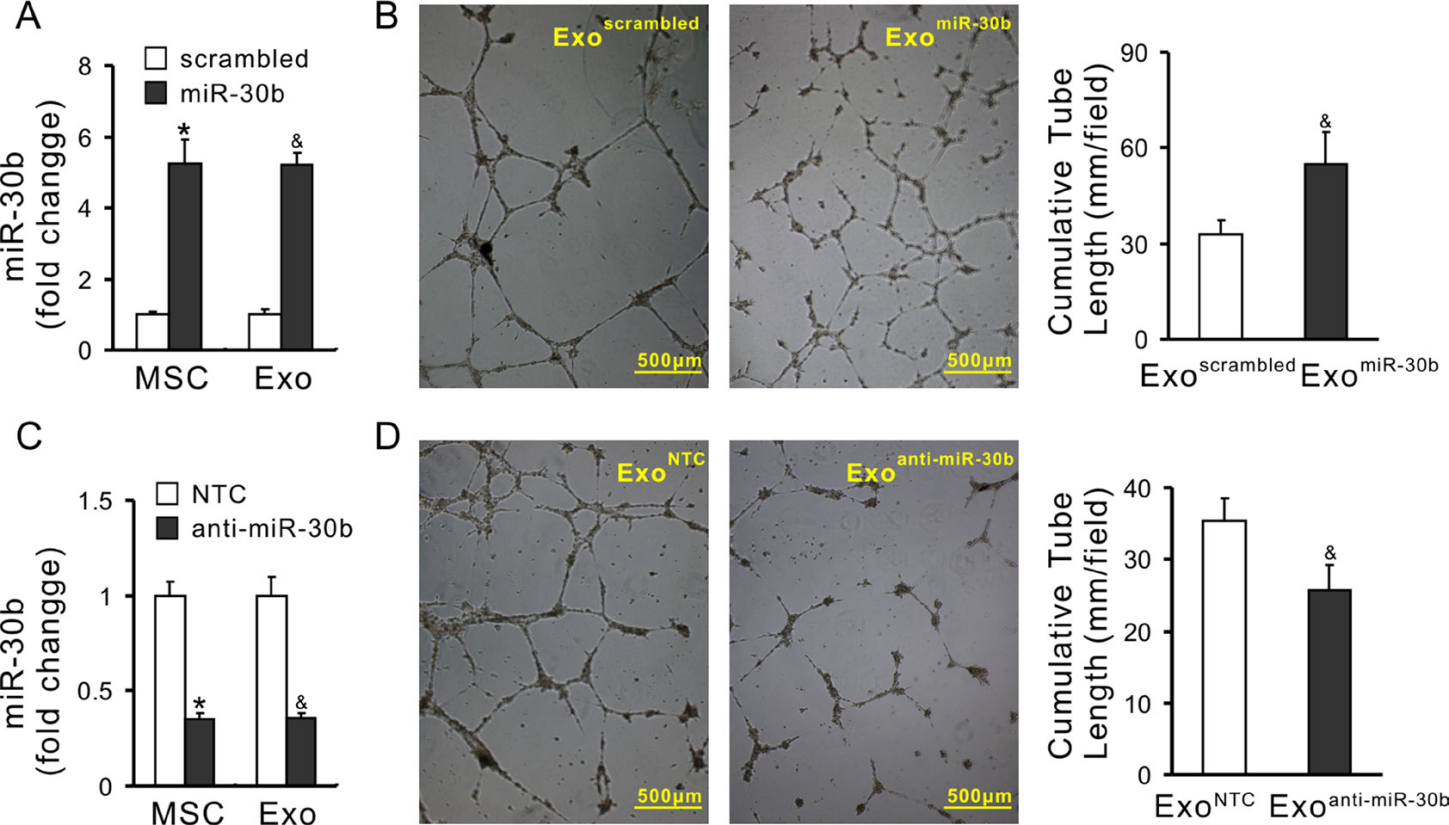
Pro-angiogenic properties of exosomes is associated with the expression of miRs (**A**) The expression of miR-30b in MSC^miR-30b^ (*P < 0.05 *vs* MSC^scrambled^) and in exosomes derived from MSC^miR-30b^ (Exo^miR-30b^) (^&^, *P* < 0.05 *vs* Exo^scrambled^); (**B**) Representative images of capillary-like structures and quantitative analysis of the total tube length (4× magnification microscopic fields) (^&^*P* < 0.05 *vs* Exo^scrambled^); (**C**) The expression of miR-30b in MSC^anti-miR-30b^ (*P < 0.05 *vs* MSC^NTC^) and in Exo^anti-miR-30b^ (^&^*P* < 0.05 *vs* Exo^NTC^); (**D**) Representative images of tube-like structures in HUVECs and quantitative analysis of the total tube length (4× magnification microscopic fields) (^&^*P* < 0.05 *vs* Exo^NTC^).

**Figure 7:**
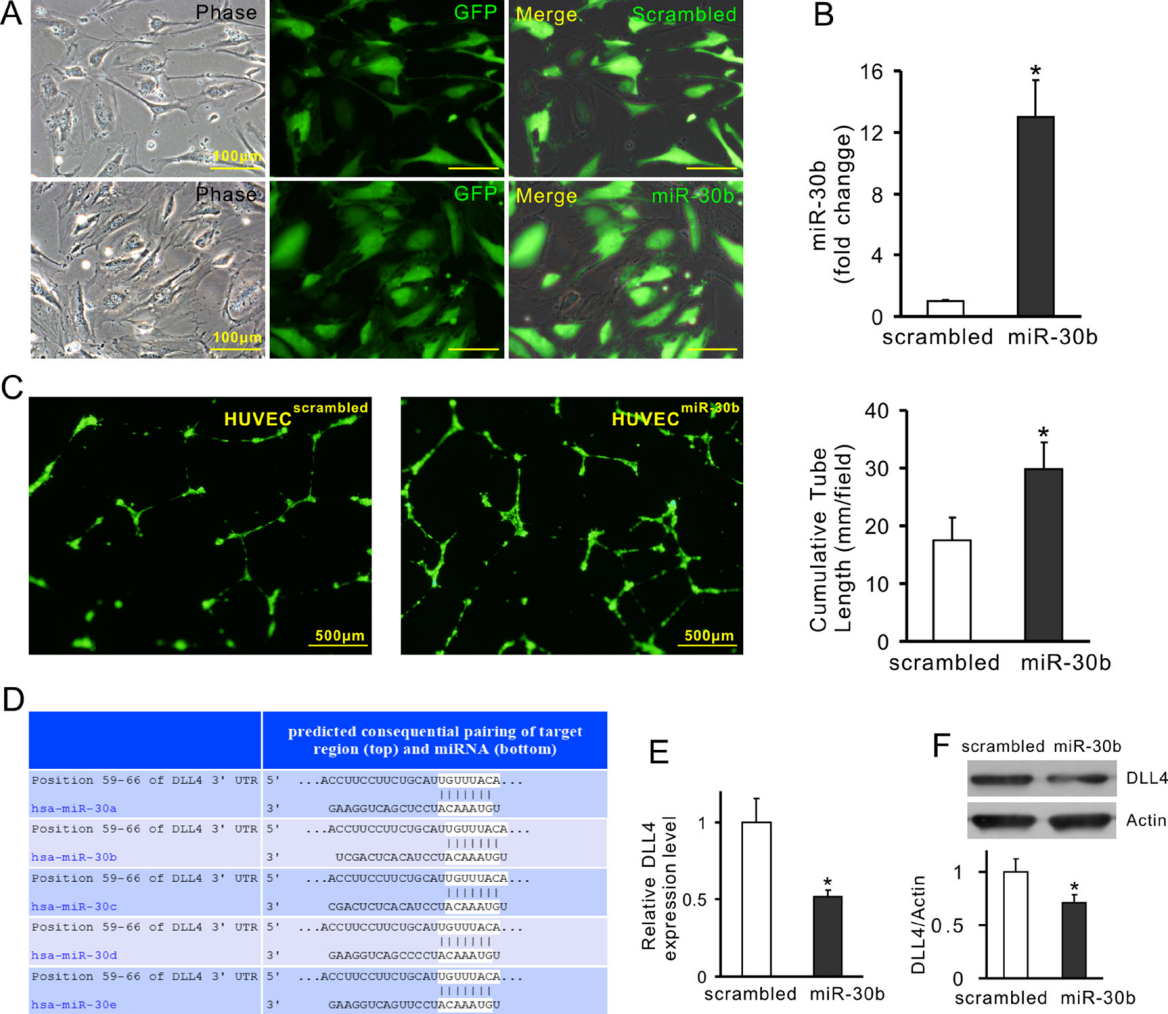
Overexpression of miR-30b downregulates DLL4 expression in HUVECs (**A**) Representative images of HUVECs with miR-30b overexpression (HUVEC^miR-30b^) and their negative control (HUVEC^scrambled^); (**B**) The expression of miR-30b; (**C**) Representative images of tube-like structures and quantitative analysis of the total tube length (4× magnification microscopic fields); (**D**) TargetScan shows that 3′ UTR of DLL4 contains conserved miR-30 family binding sites; (**E** and **F**) The expression of DLL4 in HUVECs (mRNA and protein, respectively) (*P < 0.05 *vs* HUVEC^scrambled^).

To demonstrate the effect of transferred miRs on angiogenesis, miR-30b was selected as a representative miR. Exosomes were obtained from MSCs in which miR-30b was overexpressed or knockdown, respectively. MSCs were infected with lentivirus carrying the pre-miR-30b fragment (MSC^miR-30b^). The expression of miR-30b in MSC^miR-30b^ and exosomes derived from MSC^miR-30b^ (Exo^miR-30b^) was 5.24-fold and 5.22-fold increase compared with their counterpart MSC^scrambled^ and Exo^scrambled^, respectively (Figure 6A). The cumulative tube length was increased in HUVECs treated with Exo^miR-30b^ (54.98 ± 9.89 mm/field) compared to HUVECs treated with Exo^scrambled^ (32.81 ± 4.68 mm/field), indicating that overexpression of miR-30b enhanced the pro-angiogenic capacity of exosomes (Figure 6B). In another set of experiment, miR-30b was downregulated using synthetic anti-miR-30b in MSCs (MSC^anti-miR-30b^). The expression of miR-30b in MSC^anti-miR-30b^ and exosomes derived from these cells (Exo^anti-miR-30b^) was significantly reduced, compared with their counterpart MSC^NTC^ and Exo^NTC^, respectively (Figure 6C). The cumulative tube length was significantly decreased in HUVECs treated with Exo^anti-miR-30b^ (25.68 ± 3.49 mm/field) compared to those treated with Exo^NTC^ (35.42 ± 3.01 mm/field) (Figure 6D), indicating that downregulation of miR-30b reduced the pro-angiogenic capacity of exosomes. Finally, HUVECs were directly infected with miR-30b (HUVEC^miR-30b^) using lentivirus carrying pre-miR-30b fragment (Figure 7A). The expression of miR-30b in HUVECs was verified by real-time PCR (Figure 7B). The cumulative tube length was increased in HUVEC^miR-30b^ (29.88 ± 4.51 mm/field) compared to that in HUVECs infected with scrambled-miR (HUVEC^scrambled^, 17.61 ± 4.28 mm/field) (Figure 7C). TargetScan shows that the 3′ UTR of DLL4 contains the conserved miR-30 family binding sites (Figure 7D). The expression of DLL4 in HUVEC^miR-30b^ was significantly reduced, compared to that in HUVEC^scrambled^ (Figure 7E and 7F).

## DISCUSSION

In this study, we investigated whether the paracrine mechanism by which MSCs promote angiogenesis is associated with exosome-mediated miRs transfer. Our data indicate that: 1) The conditioned medium of MSCs significantly increased tube-like structure formation, spheroid-based sprouting and neo-angiogenesis in Matrigel plug; 2) Exosomes derived from MSCs mediated the transfer of miRs from MSCs to HUVECs and promoted angiogenesis; and 3) Gain-and-loss function of miRs in exosomes demonstrated that the pro-angiogenic effect of exosomes is dependent on their pro-angiomiRs cargo.

MSCs have been considered as ideal candidates for cell-based pro-angiogenic therapy. It has been identified that MSCs secrete a variety of bioactive components including proteins, cytokines, chemicals, etc. [33, 34]. Our results demonstrated that conditioned medium of MSCs promoted HUVEC mediated tube-like structure formation, increased sprouts from HUVECs spheroids, attracted endothelial cell migration, and promoted endothelial cell proliferation (Supplementary Figure 2). These findings that MSCs promote angiogenesis through paracrine mechanisms are consistent with previous reports [35, 36].

Several miRs have been considered to participate in the regulation of various aspects of angiogenesis, including proliferation, migration and morphogenesis of endothelial cells [30, 31, 37, 38]. Pro-angiomiRs promote angiogenesis by targeting different regulators in angiogenic signaling pathways [29]. In this study, the expression of 26 pro-angiomiRs was quantified in CdM^MSC^ and screened out that miR-30b, miR-30c, miR-424 and let-7f were implicated in MSC-mediated angiogenesis. Moreover, the expression of these miRs in HUVECs was significantly increased following treatment with CdM^MSC^. All of these results indicate that the extracellular pro-angiomiRs derived from MSCs indeed transferred into HUVECs. Therefore, the angiogenetic effects of MSCs may be related to the secretion of pro-angiomiRs and transfer of these miRs into endothelial cells.

Recently, it has been shown that exosomes secreted from MSCs play a critical role in MSC-mediated paracrine effects via transfer of miRs [39]. Extracellular miRs exist with remarkable stability in various types of body fluids and cell culture media in the vesicle-associated form [40]. To confirm whether exosomes act as a vehicle for miRs carriage and transfer, we first used GW4869 to inhibit the exosomes secretion. GW4869 controls ceramide synthesis and regulates the secretion of exosomes [32]. Our results indicated that GW4869 significantly reduced the secretion of pro-angiomiRs from MSCs into culture medium. The expression of these pro-angiomiRs was also reduced in HUVECs treated with CdM^GW4869^. Moreover, the pro-angiogenic capacity of CdM^MSC^ was reduced after inhibiting or depleting exosomes in the CdM (Supplementary Figure 3), which was consistent with previous reports [41, 42]. These results revealed that the angiogenic effect of CdM was at least partly attributable to exosomes and that inhibition of exosome secretion decreased the transfer of miRs resulting in inhibition of angiogenesis.

The pro-angiogenic capacity of exosomes derived from MSCs was directly investigated through *in vitro* and *in vivo* studies. It is reported that exosomes contain plenty of miRs, including pro-angiomiRs (miR-21, miR-126, miR-130a, miR-132, miR-210, miR-378 and let-7f, etc.) [21, 43, 44]. To determine the role of transferred pro-angiomiRs in angiogenesis, we selected miR-30b amongst the cluster of miRs (miR-30b, 30c, 424 and let-7f) discovered in this study for gain-and-loss function in MSCs. Infecting MSCs with miR-30b not only upregulated the expression of miR-30b in MSCs, but also increased its expression in exosomes derived from these cells. Treatment of HUVECs with these exosomes significantly promoted tube-like structure formation. In contrary, inhibiting expression of miR-30b in exosomes resulted in reduced angiogenesis. These results indicate that miR-30b carried by exosomes plays an important role in MSCs mediated angiogenesis. Previous studies reported that DLL4, one of miR-30 family targets, modulates endothelial cell behavior during angiogenesis [31, 45]. DLL4 is a membrane-bound ligand belonging to the Notch signaling family and negatively regulates vascular sprouting and vessel branching [46, 47]. Our study showed that DLL4 expression in HUVECs^miR-30b^ was significantly reduced. It has been noted that there are many other pro-angiogenic miRs in the MSC-exosomes besides 26 miRs mentioned here. It is possible that these pro-angiogenic miRs may play some roles in angiogenesis. In addition, exosomes derived from MSCs contain many growth factors, cytokines, and chemokines which may also participate in angiogenesis.

In this study, we used medium and BSA as control for CdM^MSC^ and exosomes, respectively. It is well known that the CdM and the contents of exosomes are much more complicated than a simple medium or BSA. Therefore a CdM from a non-MSC cell line would be more appropriate for comparison. However, it is difficult to screen one suitable non-MSC cell as control cell, since the contents of CdM and exosome derived from other cells are also very complex. They may contain different pro-angiogenic factors or other factors, which may variably affect the angiogenesis more or less. It is noted that several previous studies also used basal medium as a control in their studies. van Balkom BW et al. [48] included basal medium as control to CdM derived from human microvascular endothelial cell line. Zhang B et al. [49] used PBS as control for exosomes derived from human umbilical cord mesenchymal stem cells and Wang X et al. [50] used medium as control for exosomes derived from cardiomyocytes.

It is well established that MSCs have a pro-angiogenic effect. However, from a translational perspective, exosomes derived from MSCs have shown encouraging therapeutic effects in various animal models as well in clinical trials [51]. The use of viable cells carries inherent risks such as microvasculature obstruction, transdifferentiation into inappropriate cell types, immune rejection, proarrhythmic side effects, and ossifications and/or calcifications [52]. Exosomes derived from MSCs eliminate many of these concerns associated with using live cells. In addition, the manufacture of cell-based therapeutics is also a challenging technology [52]. In contrast, exosomes can be easily stored at −20°C for 6 months without loss of biological activity [53]. Therefore, exosome-based, cell-free therapies in regenerative medicine can be easier to manufacture and prima facie safer [53].

In conclusion, our studies suggest that the MSCs promote angiogenesis via secreting exosomes that deliver pro-angiomiRs and regulate their targets in recipient cells. MSC-derived exosomes could be considered for using in therapeutic angiogenesis especially for ischemic diseases.

## MATERIALS AND METHODS

### Animals

The University of Cincinnati Institutional Animal Care and Use Committee pre-approved all animal experiments. C57BL6 mice were housed under specific pathogens free (SPF) laboratory conditions and maintained under optimal temperature, humidity, photoperiods (12L: 12D) with food and water ad libitum.

### Concentration of conditioned medium (CdM) and collection of exosomes

MSCs were first seeded at 3 × 10^6^ per 15-cm plate in complete DMEM/F12 medium for 24 h. Then the medium was replaced with 15 ml of serum-free medium. After being cultured for 48 h, the medium was collected and centrifuged to remove cell debris. The supernatant was filtered through a 0.45-mm PVDF filter (Millipore) and centrifuged 3,200 g at 4°C for 45 minutes, and then transferred into ultra-filtration conical tubes (Thermo Scientific^™^ Pierce) to concentrate medium to 100×.

Exosomes were isolated from concentrated CdM using an ExoQuick-TC Exosome Precipitation Solution (System Biosciences) following instructions provided in the manual [54]. Exosome pellets were resuspended with DMEM medium and stored at −80°C for use. The quantity of exosomes was expressed as exosome-associated proteins, using the BCA method (Thermo Fisher Scientific). The quality of exosomes was confirmed by dynamic light scattering using a particle and molecular size analyzer (ZetasizerNano ZS, Malvern Instruments) according to the manufacturer's instructions. The expression of CD9, CD63 and HSP70 was quantified using western blotting.

The morphology of exosomes was observed under transmission electron microscope (JEOL JEM-1230) as described previously [54]. In brief, exosomes were fixed with 2% paraformaldehyde and 2% glutaraldehyde in 0.1 M sodium cacodylate buffer at pH 7.3. The sample was then loaded on a carbon-coated electron microscopy grid and stained with 2% methylamine tungstate for 45 second and air-dried before exosome samples were observed in a JEM-1230 electron microscope.

### Endothelial cell proliferation assay

HUVEC proliferation was determined using a MTS assay (CellTiter 96 AQueous One Solution Cell Proliferation Assay Kit, Promega), following the manufacturer's instructions. HUVECs were purchased from ATCC (Manassas, VA, USA) and cultured in endothelial cell growth medium (ECGM) (Cell Applications). HUVECs were seeded into 96-well plates at an initial density of 2 × 10^3^ cells/well and incubated at 37°C for 12 h. After synchronization with 2% FBS for 24 h, cells were co-cultured with CdM^MSC^ for 24, 48, 72, and 96 h, respectively. A curve of cell proliferation was constructed by measuring cell growth with a microplate M3 spectrophotometer (Molecular Devices) at 490 nm.

### Angiogenesis models

Three angiogenic models were used in this study: 1) Tube-like structure formation: HUVECs (3 × 10^4^ cells/well) were seeded on top of Matrigel (BD Biosciences) in a 24-well plate and treated with the CdM (CdM-fresh medium, 1:1) or exosomes (100μg/ml) for 16 h. Images were taken on a phase-contrast microscope (Olympus), and the cumulative tube length of the network structure was quantified by randomly selecting five microscopic fields (4× magnification) using Image J software. 2) Spheroid-based sprout: Endothelial-cell spheroids were generated as described previously [55] with minor modifications. In brief, GFP^+^ HUVECs (500 cells/spheroid) were suspended in ECGM containing 0.2% carboxymethylcellulose (Sigma) and seeded in non-adherent round-bottom 96-well plates (Greiner) overnight. Spheroids were generated and embedded into Matrigel for 16 h in the presence of CdM (CdM-fresh medium, 1:1), or endothelial cell serum free defined medium (Cell Applications) only. Images were taken with an Olympus digital camera under Olympus BX 41 microscope. Capillary sprouting was quantified by measuring the cumulative length using Image J software from 10 to 15 spheroids. 3) Matrigel plug assay: Matrigel plug was performed as described previously [41], with minor modifications. Matrigel (BD Biosciences, 500 μl) containing 15 U of heparin (Sigma) was mixed with DMEM, CdM (derived from 3 × 10^6^ MSCs) or exosomes (100μg/plug). C57BL6 mice (6- to 8-week-old) were anesthetized with ketamine/xylazine (100/10 mg/kg, IP) and then subcutaneously injected with Matrigel along the abdominal midline. After 2 weeks, animals were sacrificed using over dose of anesthetic. Plugs were excised, embedded in O.C.T. and sections were cut at 7 μm. The infiltration of endothelial cells was determined by immunostaining for CD31. The images were photographed using an Olympus BX 71 microscope equipped with a digital camera (Olympus). Image-J software was used to measure CD31 positive cells in each field. The hemoglobin content of the Matrigel plugs was determined using Drabkin's reagent kit (Sigma, St Louis, MO) after the Matrigel plug was homogenized in deionized water and centrifuged to obtain the supernatant. The standard curve was generated using Stanbio^™^ Cyanmethemoglobin Standard (Stanbio Laboratory, Boerne, TX).

### Non-contact cell co-culture

MSCs were non-contact co-cultured with HUVECs using Corning^®^ Transwell^®^ (75 mm polycarbonate membrane cell culture insert, 0.4 μm pore; Corning Inc). HUVECs were seeded onto the bottom of the plate at 1 × 10^6^ per dish and cultured in ECGM. MSCs were seeded and pre-cultured onto the insert at 1 × 10^6^. The next day, the insert was placed into the plate pre-cultured with HUVECs. The medium was replaced by serum-free DMEM medium for 48 h. The culture medium was collected and concentrated into 100×. In addition, MSC-MSC and HUVEC-HUVEC co-culture were prepared as parallel control groups, respectively.

### Internalization of exosomes

Exosomes were labeled using PKH26 (red) according to manufacturer's instructions (Sigma-Aldrich) [54] and added into the HUVEC culture at a concentration of 100 μg/ml. The plate was placed into the IncuCyte ZOOM Live Content Imaging System (ESSEN BIOSCIENCE) installed inside an incubator and recorded every 2 h for 12 h.

### Real-time PCR

Total RNA was isolated from concentrated CdM, exosomes, MSCs, and HUVECs using miRNeasy Micro Kit (Qiagen). Briefly, 200 μl CdM, exosome pellet or cell pellet was lysed by 1 ml QIAzol Lysis Reagent. After 5 min incubation for homogeneity, 3.5 μl of Spike-In control (ce-miR-39, 1.6 × 10^8^ copies/μl working solution, Qiagen) were added, mixed thoroughly, and shaken for 15 seconds and then 200 μl of chloroform were added. The mixture was centrifuged for 15 minutes at 12,000 × g at 4°C following incubation for 2 min. Subsequent upper aqueous phase extraction and filter cartridge work were performed following manufacturer's instructions. cDNA synthesis and quantitative PCR was performed using miScript PCR Starter Kit (Qiagen). In brief, 1 μg total RNA from each preparation was used for the first-strand cDNA reverse transcription in the 20 μl system. Real-time PCR was performed with specific primers in iQ5 real-time PCR system (Bio-Rad) according to miScript SYBR Green PCR Starter Kit's recommendations. Data were normalized to results obtained with primers specific for U6 or in case of CdM and exosomes using synthetic Spike-In control (Ce-miR-39) as an internal control.

### Overexpression and knockdown of miR-30b in MSCs and HUVECs

A lentiviral system was used to attain effective overexpression of miR-30b in MSCs and HUVECs. The pre-miR-30b-copGFP expression plasmid and scramble-copGFP control were purchased from System Biosciences Company. miR-30b plasmid or scrambled plasmid and packaging plasmids were co-transfected into 293Ta cells according to manufacturer's instruction for production of high titer lentiviral particles. MSCs and HUVECs were infected with the generated high titer lentiviral particles for 24 h. The cell pools were selected by puromycin after several infection times. In addition, synthetic anti-miR-30b (Ambion) was transfected into MSCs using Lipofectamine™ RNAiMAX (Invitrogen) to downregulate the expression of miR-30b in MSCs. The synthetic negative miR was used as control.

### Western blotting

Western blotting was performed as described previously [56]. Briefly, cells and exosomes were lysed by RIPA buffer containing PMSF (Qiagen) and quantified with BCA protein assay reagent (Thermo Fisher Scientific). Approximately 30 μg of total protein per lane were loaded. After electrophoresis, proteins were transferred to polyvinylidene fluoride membranes (Millipore). All membranes were then blocked with 5% skim milk in TBST at room temperature for 1 h and incubated with the following primary antibodies: anti-CD9 (Abcam), anti-CD63 (Applied Biological Materials), anti-HSP70 (Cell Signaling) and anti-DLL4 (Millipore), at 4°C overnight. After washing three times with TBST, the membranes were probed with appropriate HRP-conjugated secondary antibodies (Cell Signaling) at room temperature for 1 h and visualized using ECL Plus kit (GE Healthcare).

### Statistical analysis

All data of *in vitro* experiments were obtained from at least three independent experiments. In *in vivo* study, more than 6 samples were used in each group. The results were presented as Means ± SD unless otherwise indicated and were analyzed using SPSS 18.0 software. Statistical analyses were performed using a two-tailed Student *t*-test or one-way ANOVA with post-hoc tests to determine significant differences between groups. *P* < 0.05 was considered statistically significant.

## SUPPLEMENTARY MATERIALS FIGURES AND TABLES


