# Supplementary material for: Mesenchymal stem cells release exosomes that transfer miRNAs to endothelial cells and promote angiogenesis
Source: Oncotarget. 2017 Apr 1;8(28):45200–12. doi: 10.18632/oncotarget.16778 (PMC5542178; doi:10.18632/oncotarget.16778)
Supplement: Supplementary file 1 [file oncotarget-08-45200-s001.pdf]

Mesenchymal stem cells release exosomes that transfer miRNAs to endothelial cells and promote angiogenesis

Supplementary Materials

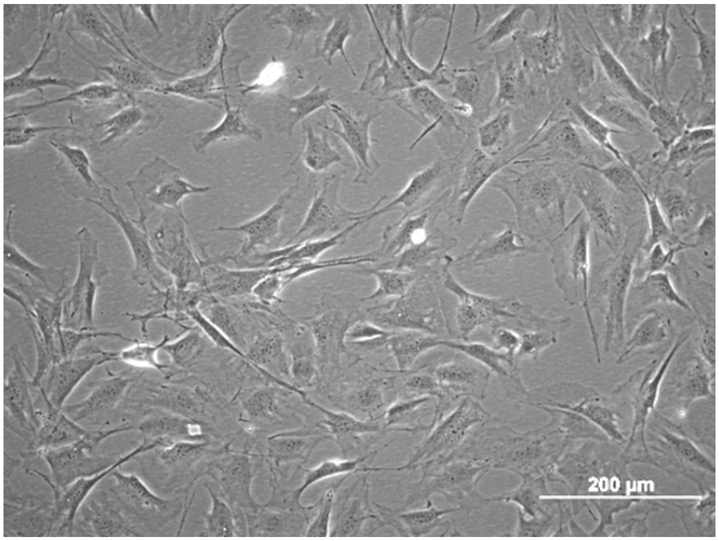

Supplementary Figure 1: Morphology of MSCs photographed under microscope. It exhibited a spindle-shaped fibroblast-like morphology.

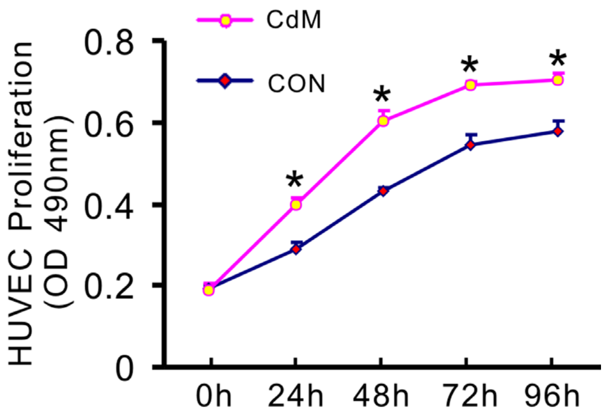

Supplementary Figure 2: CdM derived from MSCs promotes the proliferation of HUVECs. MTS assay in HUVECs treated with or without CdM^{MSC} (*, $P < 0.05$ *vs* CON).

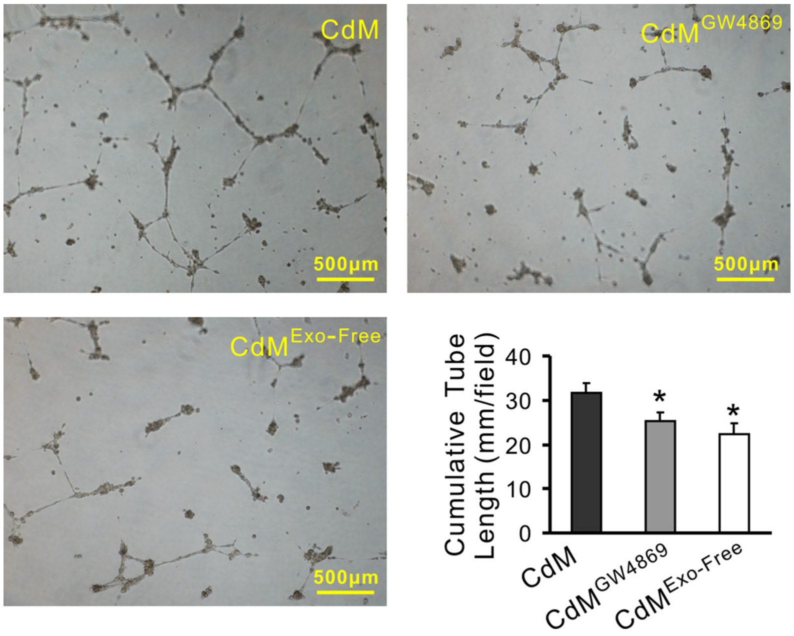

Supplementary Figure 3: The tube-like structure formation of HUVECs was reduced after inhibiting or depleting the exosomes from the CdM. Representative images of tube-like structures and quantitative analysis of the total tube length (4× magnification microscopic fields) ($*P < 0.05$ vs CdM^{MSC}).

Supplementary Table 1: The expression of proangiogenic miRNAs in CdM^{MSC} after adding into HUVECs culture for 48 h (*$P < 0.05$ *vs* CdM^{MSC})

Downregulated		
**miRNA**	**CdM^{MSC} $2^{(-\Delta Ct)}$**	**CdM^{MSC} with HUVECs $2^{(-\Delta Ct)}$**
miR-296-3p	1.076 ± 0.026	0.166 ± 0.015*
miR-378	0.572 ± 0.078	0.239 ± 0.028*
miR-18a	0.063 ± 0.006	0.036 ± 0.002*
miR-296-5p	0.060 ± 0.005	0.003 ± 0.001*
miR-9	0.0034 ± 0.0006	0.0013 ± 0.0003*

Upregulated		
**miRNA**	**CdM^{MSC} $2^{(-\Delta Ct)}$**	**CdM^{MSC} with HUVECs $2^{(-\Delta Ct)}$**
miR-17	0.513 ± 0.052	1.910 ± 0.197*
miR-20a	0.445 ± 0.048	1.503 ± 0.213*
miR-93	0.506 ± 0.092	1.266 ± 0.188*
miR-132	0.060 ± 0.013	0.307 ± 0.081*

No significant differences		
**miRNA**	**CdM^{MSC} $2^{(-\Delta Ct)}$**	**CdM^{MSC} with HUVECs $2^{(-\Delta Ct)}$**
miR-23a	24.220 ± 4.602	19.767 ± 0.097
miR-23b	21.017 ± 2.057	17.646 ± 1.037
miR-27a	38.411 ± 3.759	39.553 ± 1.744
miR-27b	28.133 ± 3.028	30.699 ± 0.602
miR-92a	4.653 ± 0.704	3.959 ± 0.019
miR-130a	5.464 ± 0.027	5.666 ± 0.444
miR-210	0.580 ± 0.003	0.597 ± 0.020
